# The impact of having a baby with cleft lip and palate on parents and on parent-baby relationship: the first French prospective multicentre study

**DOI:** 10.1186/s12887-020-02118-5

**Published:** 2020-05-18

**Authors:** Bruno Grollemund, Caroline Dissaux, Pascale Gavelle, Carla Pérez Martínez, Jimmy Mullaert, Toni Alfaiate, Antoine Guedeney, CLIP team (Cleft Lip & palate Infant Parent), M. P. Vazquez, C. Bruand-Rodier, A. Danion-Grilliat, A. Picard, V. Soupre, E. Galliani, F. Zazurca, P. Pellerin, V. Martinot-Duquesnoy, C. Frochisse, B. Kabuth, J. Y. Gall, I. Kauffmann, M. Barriere, G. Corduan, M. Velten, J. Jegu, Hélène Kuissu, Stéphane Hecketsweiler, Hanan Idrissi, Aurélie Kosmala, Catherine Forter, Michèle Brute

**Affiliations:** 1 Département d’Orthopédie Dento-Faciale, Pôle de médecine et chirurgie buccodentaires, Cleft Competence Center, Strasbourg University Hospital, Place de l’Hôpital 1, 67000 Strasbourg, France; 2 Maxillofacial and Plastic Surgery Department (Head: Prof. C. Bruant-Rodier), Cleft Competence Center, Strasbourg University Hospital, 1 place de l’Hôpital Civil, 67091 Strasbourg, France; 3 Hôpital Necker Enfants malades, Paris France. Service de chirurgie maxillo-faciale et plastique. Centre de référence des fentes et malformations faciales, Hôpital Necker Enfants Malades, Paris, France; 4 Cambrigde, MA USA; 5 Département d’Epidémiologie, Biostatistique et Recherche Clinique, Unité de Recherche Clinique HUPNVS Hôpital Bichat - Claude-Bernard, Paris, France; 6 Département d’Epidémiologie, Biostatistique et Recherche Clinique, Unité de Recherche Clinique HUPNVS; INSERM CIC-EC, 1425 Paris, France; 7 HUPNVS Hôpital Bichat - Claude-Bernard, Univ Paris Denis Diderot, CESP Inserm U 1178 et LPPS, 4057 Paris, EA France

**Keywords:** Cleft lip, Cleft lip and palate, Parental representation, Parenting stress, Prenatal diagnosis, Psychosocial parent-infant relationship, Relational development, Social withdrawal behaviour, Repair surgery, EPDS, IOFS, ADBB, PSI

## Abstract

**Background:**

The objective of this prospective, multidisciplinary and multicenter study was to explore the effect of a cleft lip, associated or not with a cleft palate, on parents, on parent-infant relationship, and on the baby’s relational development. It also highlighted how the type of cleft and the timing of the surgery could impact this effect.

**Method:**

158 infants, with Cleft lip with or without Palate, and their parents participated in this multicenter prospective cohort. Clinical evaluations were performed at 4 and 12 months postpartum. The impact on the parents and on the parent-infant relationship was evaluated by the Parenting Stress Index (PSI), the Edinburgh Post-partum Depression Scale (EPDS) and the Impact-on-Family Scale (IOFS). The relational development of the infant was assessed using the Alarm Distress Baby Scale (ADBB). The main criteria used to compare the infants were the severity of cleft and the time of surgery.

**Results:**

The timing of surgery, the type of malformation or the care structure had no effect on social withdrawal behaviors of the child at 4 and 12 months postpartum (ADBB). Furthermore, early intervention significantly decreased maternal stress assessed with the PSI at 4 months. Parents for whom it had been possible to give a prenatal diagnosis were much better prepared to accept the waiting time between birth and the first surgical intervention (IOFS). Higher postpartum depression scores (EPDS) were found for both parents compared to the general population.

**Conclusion:**

A joint assessment of the mental health of both infants and parents is required in the follow-up of cleft lip and palate. Even if most families are remarkably resilient faced with this major cause of stress, a significant proportion of them could require help to deal with the situation, especially during this first year of follow-up. An assessment of the child’s social withdrawal behaviour and of the parental stress and depression appears useful, in order to adapt care to infant and parent’s needs.

**Trial registration:**

ClinicalTrials.gov Identifier: NCT00993993. Registered 10/14/2009 <.

## Background

Cleft Lip and Palate (CLP) is the most frequent congenital craniofacial malformation in humans [1]. This medical condition is not a major cause of mortality in developed countries; however, it does cause considerable morbidity among children who are affected, and their family [2]. Previous studies observed a higher prevalence of psychosocial disorders of children with CLP. Tillman et al. [3] shows an increased prevalence of psychiatric illness, intellectual disability, language disorders, Autism Spectrum Disorders (ASD), hyperactivity and other behavioural disorders. In addition, difficulties in interactional skills have been observed [4]. Habersaat et al. [5] describes that babies with CLP interact less with their mother at 2 months of age, compared to controls. Other studies [6] point the fact that mothers are less responsive and less sensitive in the interpretation of their infant’s signals. Facing his child with this facial malformation directly impairs the process of becoming a parent and the parent-infant relationship [7]. Parents psychological burden starts when CLP is diagnosed, before or at birth. Parents could experience several emotional reactions, such as confusion, distress, guilt [8], loss of control, helplessness, and even depression [9]. Parents can feel damaged by their perceived inability to produce a healthy and well-being baby, free from any physical defects [10–13]. As a result, parents have to go through a grieving process, in order to accept the child’s difference [9]. The impact of the malformation on parent-infant relationship could be influenced by the type of cleft. While Endriga and Speltz [14] report that mothers of cleft palate children are more distant than the ones of cleft lip and palate children, other ones as Despars et al. [7] observe that the severity of the cleft is not related to parental representations or stess. Thus, it would be worthwhile evaluating how the type of cleft impacts the parent-infant relationship.

Surgical treatment of cleft lip and palate remains complex [15] and not standardized, as the surgery type and timing is more related to the experience of the surgeon and to the centre habits [16]. It is also suggested that lip surgical repair could affect parent-infant interactions [17]. As previous studies report early repair promote a better parent-infant interaction [8, 18], late repair could also be advantageous by giving the time the parents need to accept the malformation and plan the surgery with the medical team [18, 19].

This prospective study aims to highlight social withdrawal of children with CLP at 12 months and to identify influencing factors (type of cleft, timing of surgery, centre …). A second objective stands in describing parental stress and depression during the first year.

## Method

### Main hypothesis

The main hypothesis of this research is that the longer the time lapse before the first surgical intervention, the more likely are parental perceptions and feelings to upset the parent–child relationship and affect the harmonious development of the child.

### Secondary hypothesis

There are also two secondary hypotheses:

1) that the parents for whom it has been possible to give a prenatal diagnosis are better prepared to accept the waiting time.

2) that with time, the negative feelings of parents in the later surgery group (3 to 6 months after birth) tend to decrease and to come into line with those of parents whose children have had an early intervention, and also that the child’s distress tends to decrease.

### Participants

This study concerns a prospective cohort of 156 children with a cleft. A detailed description of the inclusion criteria, the recruiting centers, and all assessed items and scales can be found in a previous article by Grollemund et al. [16]. All parents were informed through a letter. They all signed written consent to be involved in the study, themselves and their children.

### Instruments

The different measures are thoroughly described in the previous article by Grollemund et al. [16]. These are the main summarized tools:

1) Social withdrawal behaviour of the child is assessed by the Alarm Distress Baby Scale (ADBB) on video clips recorded during follow-up consultations. Independent scoring is conducted by an expert, and by the clinician immediately after the examination. A consensus score is calculated each time there is a discrepancy between the scores given by the clinician in charge of the assessments and the expert (CPM). The highest the score gets, the worst the social withdrawal behaviour is. As soon as a score over or equal to 5 is obtained, a child behaviour withdrawal could be noticed.

2) The Parenting Stress Index (PSI) screens for parental attitudes that could be risk factors for the development of emotional and developmental disturbances in a young child. The highest the score is, the most stressful the parents could appear.

3) The Edinburgh Post-partum Depression Scale (EPDS) is completed by each parent, in its validated French version. The highest the score gets, the highest the depression level is. When EPDS score is over or equal to 11, there is a depression state. Mean rate for post-partum depression in general population is 13% at 8 months.

4) The Impact on Family Scale (IOFS) assessing the family, social and financial impact of the malformation is also realized. This instrument, initially developed by Stein et al. [20] with 33 items and 4 dimensions was progressively reduced to a 15-item questionnaire with one main dimension representing general negative social impact on the family [21, 22]. In the French validation of IOFS, the lower the score gets, the highest the family, financial and social impact is.

Results related to the presented objectives and risk factors influencing these results (timing of the surgery, severity of the malformation) are reported in this issue.

### Design

This study is a prospective cohort of children diagnosed with Cleft lip with/witout palate. Two evaluation periods are planned: T0, when the child is 4 months, and T1 when the child is 12 months, i.e. at least 6 months after the first surgical intervention. Children with cleft lip, alveolar cleft lip or cleft lip and palate are included. Isolated cleft palate forms are exluded. It involves isolated or family-related forms, either syndromic (associated with other abnormalities or malformations) or non-syndromic forms. The children and their parents come from one of the four centres involved in the study. One center realizes neonatal surgery at 1 month, one centre operates at 3 months, a third one used to operate at 1 month then changed during the time of the study to 3 months, a last one realizes the lip closure at 6 months. Parents are included following informed consent for themselves and their child. The ADBB score measured at 12 months is used as the main criterion to identify factors associated with social withdrawal.

### Statistical procedures

In the descriptive analysis, patient characteristics are described using frequencies and percentages for categorical variables, and medians and inter-quartile range values for continuous variables. Correlations between ADBB, PSI, IOFS and EPDS scores are estimated using Spearman’s rank correlation coefficient. Baseline factors associated with the ADBB score at T1 among the clinical and demographic characteristics (type of centre, time lapse to surgical intervention, diagnosis, side of the cleft, type of cleft, interaction between side and type of cleft) are identified using univariate linear models. The effect of each binary variable on the ADBB score is analysed by way of the differences of means and a 95% confidence interval is obtained by bootstrapping 10,000 samples. The authors also use the *p*-value provided by the Wilcoxon non-parametric test. Factors associated with the PSI score are studied using the same methodology.

Sample size calculation: this study is initially powered to detect a difference of 2 ± 3.78 points for the ADBB score between groups with early and late surgical intervention at 1 year, with a power of 90%, an alpha error of 5% and assuming a dropout rate of 5%. Considering these hypotheses, the total study calculated sample size is 160 patients. This study is also performed to detect a difference of 22.9 ± 41.9 points for the PSI score between the 2 groups (early and late surgical intervention) using the same parameters. A *p*-value of < 0.05 is considered statistically significant. Analyses are performed using SAS V.9.4 (SAS Institute Inc., Cary, North Carolina, USA) and R 3.4.0.

## Results

**Fig. 1:**
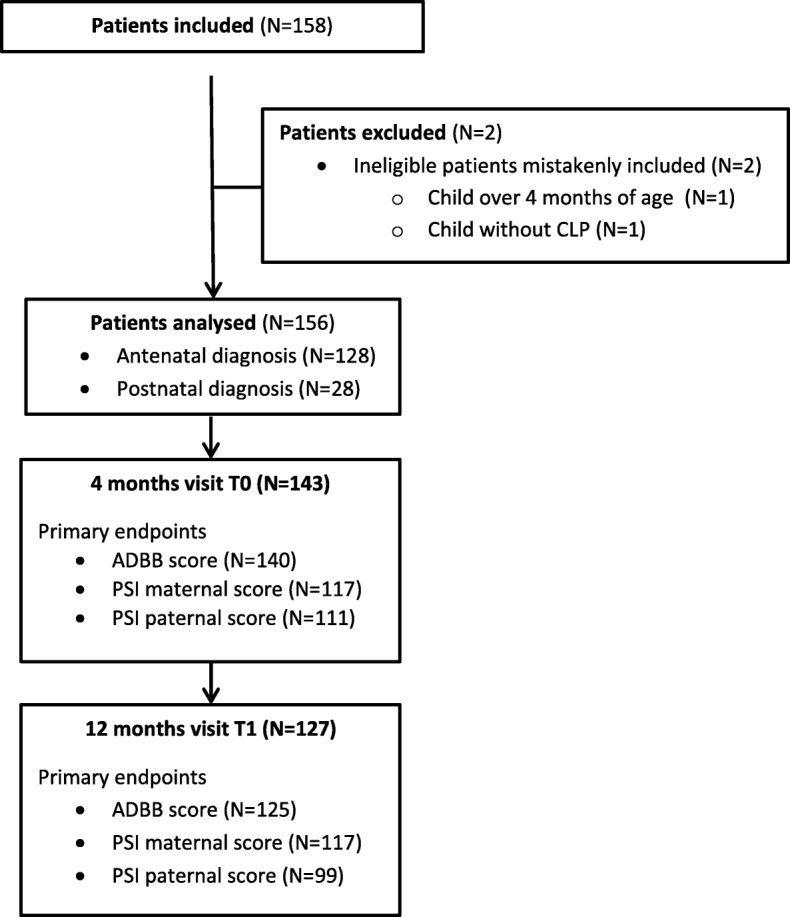
Flowchart of the study

Two children are excluded from the study (Fig. 1). One of them was over 4 months of age at T0 and the other one had no cleft. The majority of the parents took part of the evaluations at T0 and T1. Only one child had a syndromic CLP, associated with other genetic abnormalities. ADBB scores are less important at T1 than at T0 because some of the families were lost to follow-up. Several PSI questionnaires missed.

Looking at the population features (Table 1), the major cleft epidemiologic criteria are retrieved such as a majority of male and unilateral clefts. 58% of children have a complete cleft lip and palate. 18% of them still had no antenatal diagnosis whereas lip was always concerned. Mean time lapse between birth and first intervention is 3.3 months [1.2–6.8].

**Table 1:**
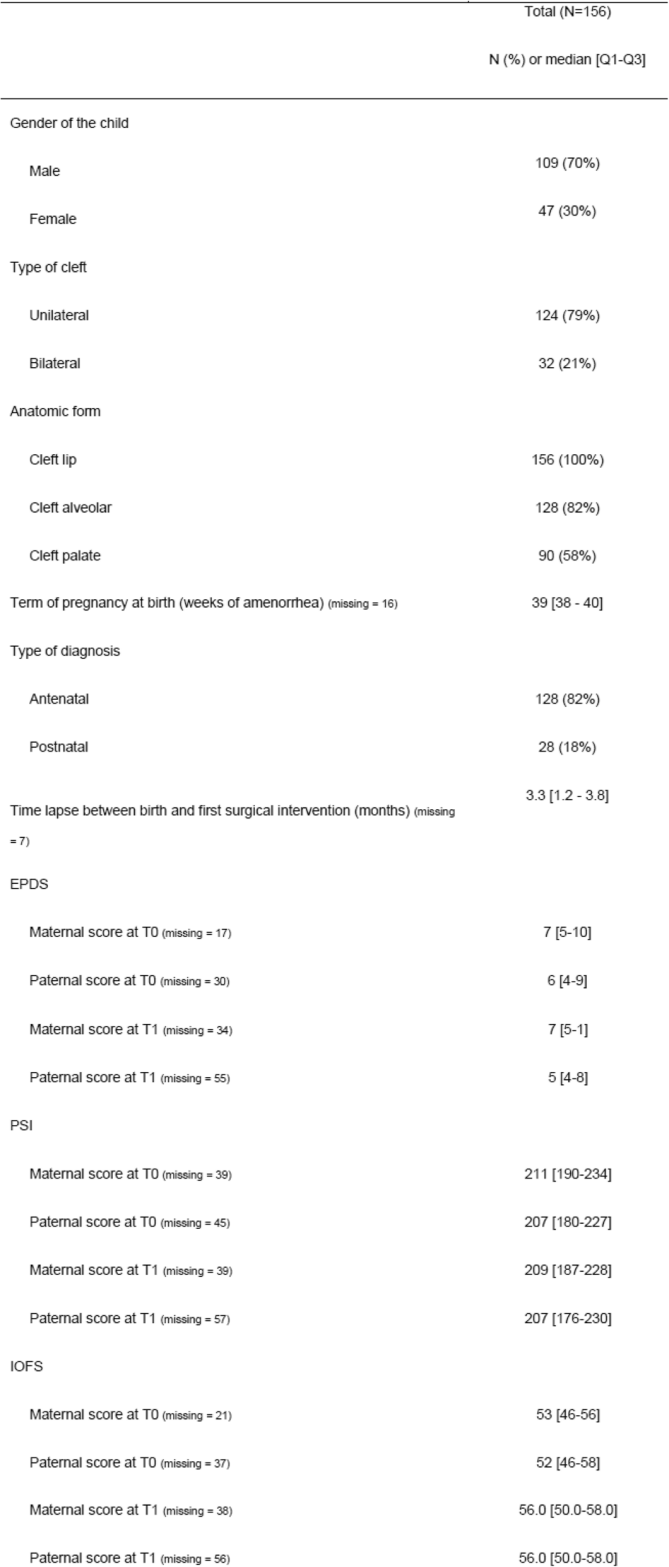
Description of the population.

Both parents are affected by this experience and show higher depression scores (EPDS) compared to the general population at T0 and T1, in the first months of treatment.

PSI scores show little significance. A lot of missing data are noticed.

IOFS scores are relatively stable between TO and T1 and can be considered equivalent between mothers and fathers.

The authors look for the influence of different factors (lack of antenatal diagnosis, timing of surgery and type of clefts considering cleft lip and bilateral cleft respectively as the least and the severe ones) on ADBB score (Table 2) or PSI (Table 3).

**Table 2:**
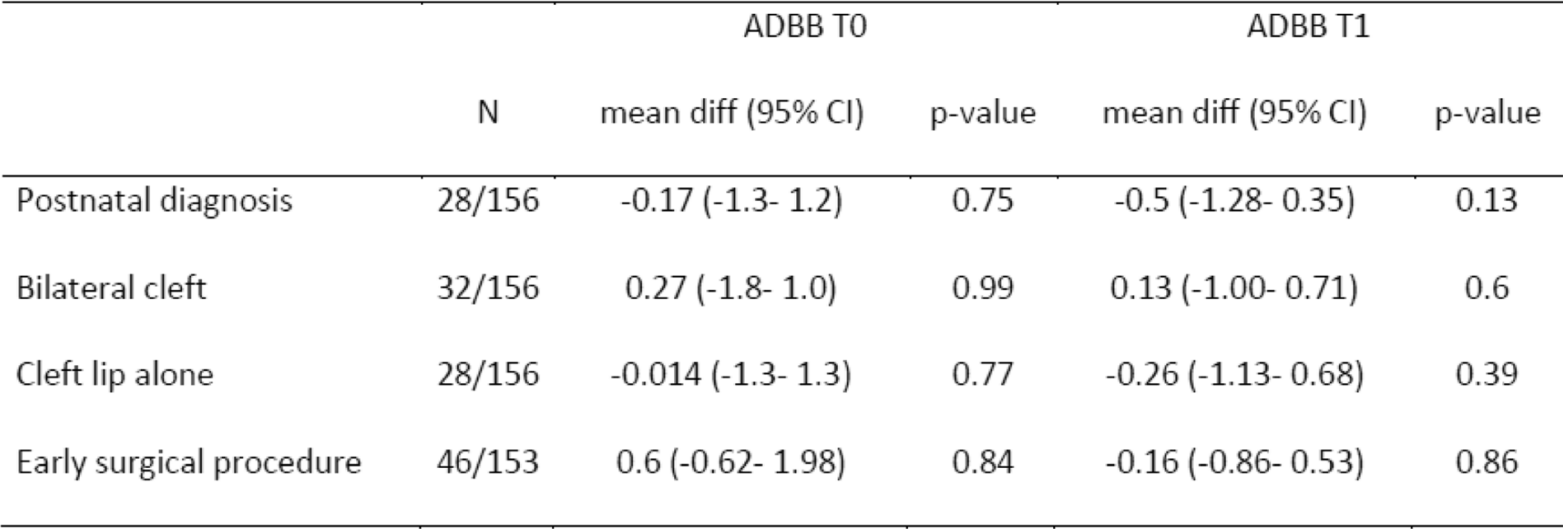
Univariate analysis. Influence of potential risk factors for social withdrawal on the ADBB score at T0 (4 months) and T1 (one year).

**Table 3:**
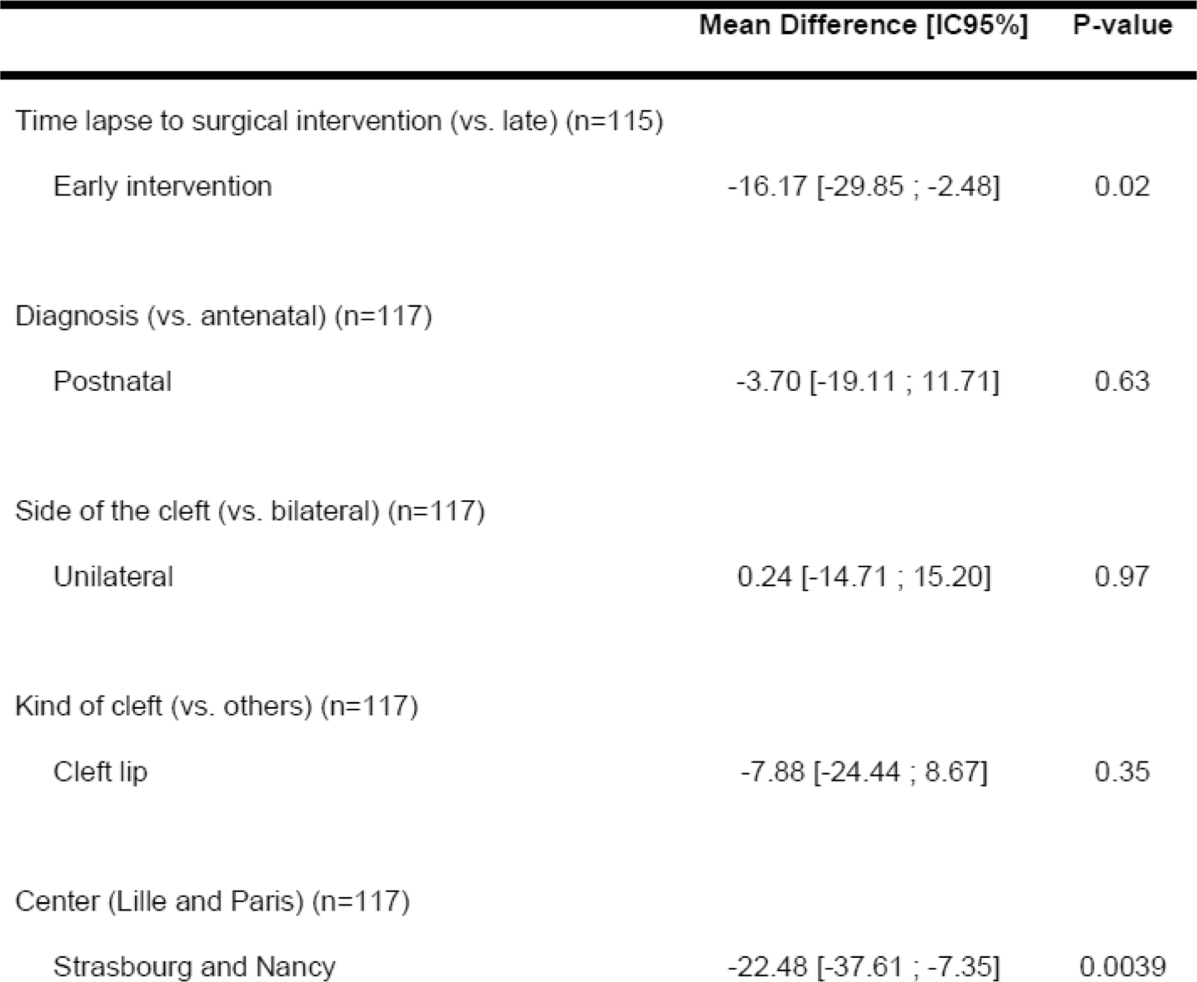
Results of the univariate analysis for PSI maternal score at T1.

In contrast with our main hypothesis that the longer the time-lapse before the first surgical intervention is, the more likely the parents’ psychological perceptions would be to affect the harmonious development of their child, there is no effect of the timing of the surgery on the social withdrawal behaviours of the child (Table 2).

There is no effect of the type of malformation on the level of social withdrawal behaviours at T0 (4 months) or T1 (12 months). The incidence of social withdrawal behaviours among CLP children (ADBB score > 5) is 13%, at 12 months of age which is the same level as that found in community studies in France.

Early intervention significantly decreases maternal stress as assessed with the PSI at 12 months (Table 3). Another association is found in between PSI score and the original centre. Smaller cleft competence centres as Strasbourg and Nancy seem less stressful for the parents than bigger ones. This result could also be related to the size of the city.

In line with the second hypothesis, parents who had been given a prenatal diagnosis were better prepared to accept the waiting time between birth and the first surgical procedure (Table 4).

**Table 4:**
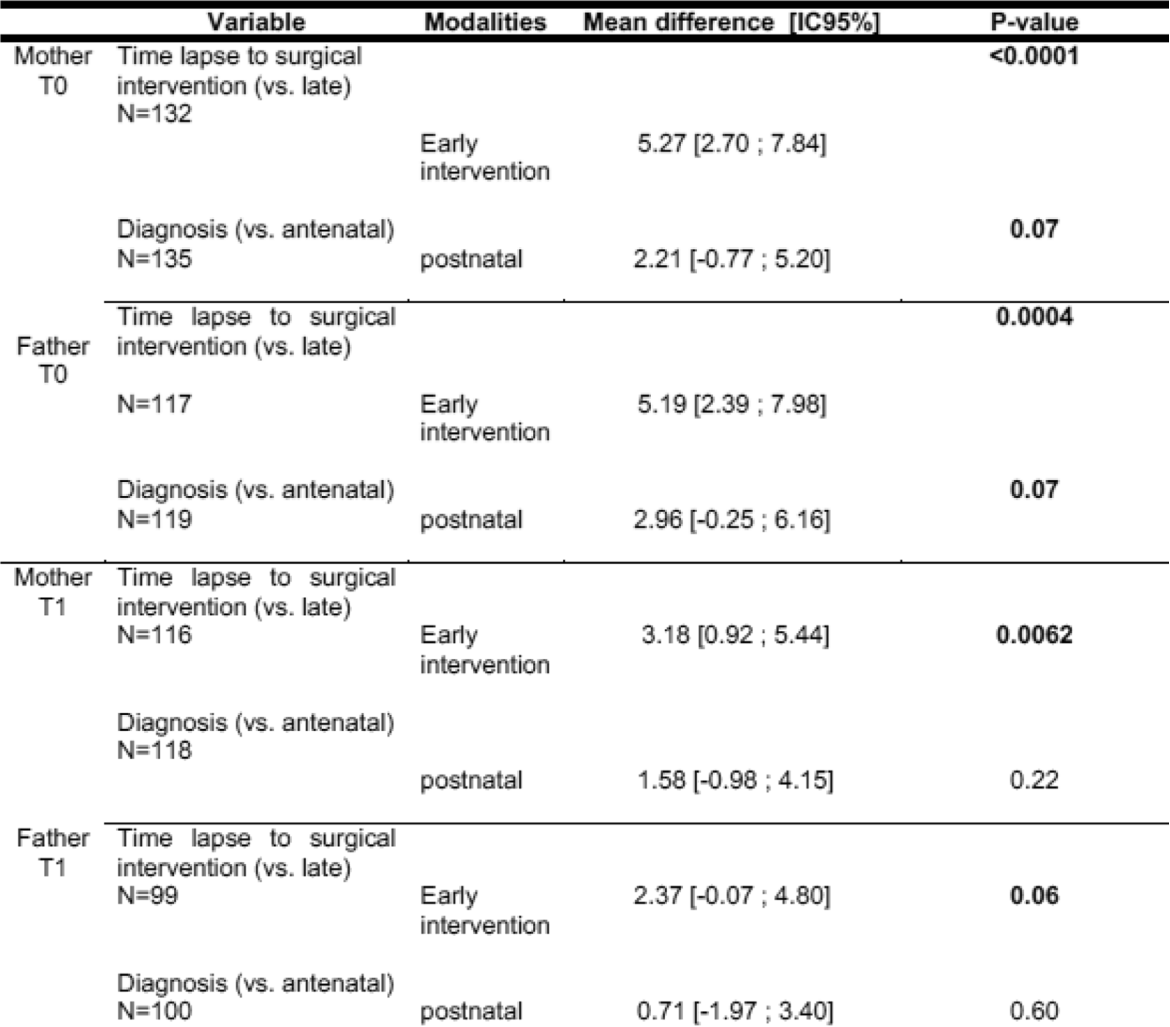
Results of the univariate analysis for EOFS of the mother and the father at T0 and T1.

**Fig. 2:**
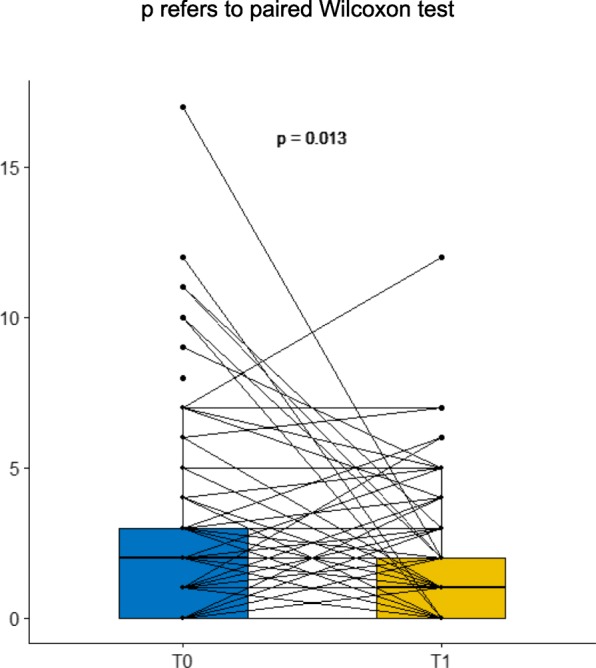
Evolution of ADBB scores between T0 and T1

Negative feelings and social impact among parents in the later surgery group (3 to 6 months after birth) tend to decrease and to come into line with those of parents whose children had an early intervention. Indeed, IOFS mean differences at T0 are statistically significant between early and late intervention groups. However the mean difference tends to decrease between T0 and T1 (5.27 to 3.18 for mothers, 5.19 to 2.37 for fathers), and for the fathers it even becomes not significant anymore at T1 (*p* = 0.06) (Table 4). Alongside, the children’s distress tends to decrease from T0 to T1, as assessed with the Alarm Distress Baby scale (Fig. 2). Even if ADBB score is at the same level as what is found in community studies, this decrease is important to consider.

**Fig. 3:**
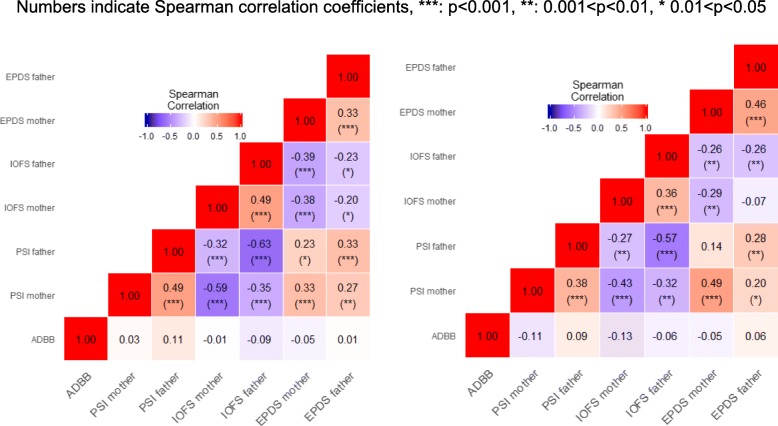
Correlation matrix between instruments evaluated at T0 (left) and T1 (right)

Figures 3 shows a heat-map of correlations between instruments administered at T0 and T1. As several PSI questionnaires are missing, the authors tend to highlight the best tools to measure cleft impact of the parent-baby relationships. This figure shows a high level of correlation, which is statistically significant, between IOFS and PSI.

## Discussion

A large array of instruments is used in this study: first ADBB scale to assess children social withdrawal behaviours, and secondly EPDS, PSI and IOFS to evaluate parents’ impact. Assessment of the child or of the parents is realized during routine follow-up consultation and so does not add any time-consuming appointment.

In literature, the relationship between the parents and the child has always been studied only by questioning the parents, most often focusing on the mother. They answer the questions on the basis of their own feelings, which can only provide one aspect of reality. The ADBB scale enables the study of the child’s withdrawal behaviours and focuses only on the child. The behaviours of an infant, and any signs of withdrawal, are unlikely to be dissimulated, while this may not be the case when the parents are questioned. To our best knowledge this is the first study on CLP using simultaneous and independent assessment of parents and infant’s mental health in such a difficult situation. The main interest of combining a direct assessment of the child and a parent evaluation is to put, for the first time, the experience of the parents after a trauma into perspective and evaluates its consequences on the child. The contribution of the ADBB scale for children in this age group enables a more objective study of the parent-child relationship, and in a more symmetrical manner.

The ADBB scale has been used in a number of problematic neonatal situations, such as the Prader Willy syndrome [23], or neonatal cardiac surgery [24], but has never been used on children with CLP. Thus, this national multicenter study is the first to use ADBB in CLP children and to show that, despite common representations, no association is found between children social withdrawal and the severity of the cleft. Interestingly, the levels of social withdrawal in these three situations (CLP, Prader-Willy, neonatal cardiac surgery) are not related to the severity of the medical condition, but rather to the level of stress and distress shown by the mothers of these infants. This result at least eliminates the likelihood of a strong negative effect and highlights the fact that infants and parents should be followed and evaluated regardless of the severity of the malformation.

These results emphasise the need to detect social withdrawal behaviours in the first months of life as a silent signal of suffering that should be interpreted and treated, especially when the infants concerned present a medical condition [24]. Along these lines, Smith-Nielsen et al. [25] suggested that adding the ADBB to existing routine developmental health follow-up practices could add value to health care workers’ practice by improving their knowledge about the socio-emotional development of infants. Even id ADBB scores are at the level of the ones found in general population, it is important to notice two main statements: EPDS is higher than general population and ADBB tends to decrease between T0 and T1. First, it highlights the fact that it is not because the child seems not secluded that the parents do not suffer and vice versa. Then, ADBB score decreases which means the child shows a distress at T0 that could be improved at T1. The surgery has probably an impact on ADBB score and on parent-infant relationship, but everything which occurs during the first year and which could impact the child comfort has to be taken into account. These babies still suffer from these first experiments of life and particular attention should be given to them and their parents.

The resilience of both children and parents is remarkable, as the level of withdrawal behaviours in this exposed population is no higher than that observed in the French community [26]. The families of children with CLP are not particularly likely to have experience psychological or psychiatric support. They may be reluctant because of certain preconceived ideas about the speciality and may argue that this consultation will not change reality and the problems they are facing. Above all they may not understand why interviews of this type could be useful. The assessment needs to be presented as an encounter enabling better acquaintance with the child, and better knowledge of any particular difficulties. In our encounters, the authors explain that if difficulties are identified, suitable care and support would be provided in a remediation process that is not only physical.

It is very difficult for parents to express their feelings using the standardised questionnaires chosen. Certain items in the PSI were unsuited to the infants’ age in this study. However numerous parents took the opportunity provided by these interviews to talk with the psychologists and psychiatrists and to confide their emotions. For some who felt particularly alone, this time for words undoubtedly provided assistance.

Given the importance of the relationship between parent and child, the clinical evaluation by surgeons should include screening of the mothers and fathers for symptoms of depression and anxiety at the time of their child’s first evaluation. This would give the clinician an opportunity to engage parents in a dialogue about the relationship between their symptoms and their child’s treatment outcomes. Even if they do not talk about it spontaneously, these “different” children and their parents do suffer, and need someone to listen to their experiences, assessing their level of stress and possible difficulties relating to the situation. Indeed, it is important not to forget the many challenges these parents will have to overcome in this first year of life: accepting the fact that they have a child with a malformation (particularly for the mothers because this came about inside their body); seeing this cleft mouth for the first time at birth; coping with the eyes of others; remaining creative and not collapsing as a parent when confronted with the refusals, failures and uncertainties of the first breast or bottle feeds; returning repeatedly to the hospital; coping with the anxiety of the first anesthesia; having to witness the child’s discomfort after surgery; having to care for their baby; and once again accepting this baby after the changes brought about by surgery. The offer of an encounter with a psychologist or psychiatrist in a surgery department is one first essential aspect. But the way in which this is presented is also important. It should not be imposed, but it should be sufficiently advocated for any parent to readily take up the offer. Findings from a recent study in United Kingdom suggest that the centralization of CL/P units has greatly enhanced patient experiences, and support the notion that psychologists should be integrated into each team [27].

While the avaibility of ADBB score is good (only 10% missing), the limitation of this study stands in the large volume of missing data concerning the secondary instruments (PSI, EPDS and IOFS).

Some parents refuse to take part in the study, which potentially leads to a selection bias in this sample. It could be thought that these refusals are mainly imputable first to the distance between home and the cleft centre, and secondly to the repeated visits required for the child’s care. This could also be the consequence of the parents’ reluctance to confide their feelings and difficulties since the discovery of the malformation. As the study could not intrude on the private lives of these families, it is impossible to press for agreement, especially in cases where refusal comes from one parent only. This applies more frequently to the father.

In literature, parent–child relationship is always studied by interviewing the parents. The choice and the relevance of the instruments used can be questioned. Indeed, self-administered measures are subject to caution, as parental responses can lack objectivity. It also raises issues concerning data of families who refuse to participate. It is likely that this seriously biases the results. Thus, it is difficult to assume that, overall, things are fairly satisfactory. Indeed, it would have been useful to know the number of families that did not take part in the study, and the reasons for their refusal, distinguishing personal reasons from practical reasons relating to travel or care centre organisation.

PSI has proven to be long and difficult for parents to fill out properly and completely, that could explain the lack of data especially on this score. Good correlations on parental scores between PSI and IOFS were found. IOFS and EPDS seems to be suitable tools to assess parental mental health during the reconstruction procedure for a cleft lip and palate, compared to PSI. This study was considered as the French validation of IOFS. This tool appears to be more useful than PSI in this particular situation. It highlights how cleft lip and palate can impair the family on a social and financial point of view.

## Conclusion

This study is the first in France to assess specifically the psychological consequences of the birth of a child with a CLP on the parents’ mental health and consequently on early parent-child relationships. The timing of surgery, the type of malformation or the care structure had no effect on social withdrawal behaviors of the child at 4 and 12 months postpartum. The resilience of both children and parents is remarkable, as the level of withdrawal behaviours in this exposed population was no higher than that observed in the French community. On the other hand, given that the consequences of parental depression on infant development are well known, this study highlights parental needs in terms of psychological support, especially during the first year of treatment when a majority of surgical steps occur. This study was the first in France gathering the French expert centres and some of the more active centres around a common protocol to explore the psychological experiences of parents and children faced with this very demanding experience of a child born with a cleft lip and palate. The results of this study have already changed some of the professional practices in the French specialized centres, adding paediatric nurse appointments to assist with problems for the baby’s feeding, to support the first mother-infant interactions, and to adjust care to the infant and the parents.

## Data Availability

The datasets used and analysed during the current study are available from the corresponding author on a reasonable request.

## Abbreviations

CL: Cleft lip malformation
CLP: Cleft lip with or without cleft palate malformation
ADBB: The alarm distress baby scale
PSI: The parenting stress index
EPDS: The edinburgh post-partum depression scale
IOFS: The impact on family scale
